# Genome-wide alternative splicing profile in the posterior kidney of brown trout (*Salmo trutta*) during proliferative kidney disease

**DOI:** 10.1186/s12864-022-08685-4

**Published:** 2022-06-16

**Authors:** Arun Sudhagar, Mansour El-Matbouli, Gokhlesh Kumar

**Affiliations:** 1 Clinical Division of Fish Medicine, University of Veterinary Medicine Vienna, 1210 Vienna, Austria; 2 Peninsular and Marine Fish Genetic Resources Centre, ICAR - National Bureau of Fish Genetic Resources, Kochi, Kerala 682 018 India

**Keywords:** RNA-seq, Post-transcriptional modification, Myxozoan, *Tetracapsuloides bryosalmonae*, Salmonids

## Abstract

**Background:**

The cnidarian myxozoan parasite *Tetracapsuloides bryosalmonae* causes chronic proliferative kidney disease (PKD) in salmonids. This parasite is a serious threat to wild and cultured salmonids. *T. bryosalmonae* undergoes intra-luminal sporogonic development in the kidney of brown trout (*Salmo trutta*) and the viable spores are released via urine. We investigated the alternative splicing pattern in the posterior kidney of brown trout during PKD.

**Results:**

RNA-seq data were generated from the posterior kidney of brown trout collected at 12 weeks post-exposure to *T. bryosalmonae*. Subsequently, this data was mapped to the brown trout genome. About 153 significant differently expressed alternatively spliced (DEAS) genes, (delta PSI = 5%, FDR *P-*value < 0.05) were identified from 19,722 alternatively spliced events. Among the DEAS genes, the least and most abundant alternative splicing types were alternative 5′ splice site (5.23%) and exon skipping (70.59%), respectively. The DEAS genes were significantly enriched for sodium-potassium transporter activity and ion homeostasis (ahcyl1, atp1a3a, atp1a1a.1, and atp1a1a.5). The protein-protein interaction network analysis enriched two local network clusters namely cation transporting ATPase C-terminus and Sodium/potassium ATPase beta chain cluster, and mixed inclusion of Ion homeostasis and EF-hand domain cluster. Furthermore, the human disease-related salmonella infection pathway was significantly enriched in the protein-protein interaction network.

**Conclusion:**

This study provides the first baseline information about alternative splicing in brown trout during PKD. The generated data lay a foundation for further functional molecular studies in PKD - brown trout infection model. The information generated from the present study can help to develop therapeutic strategies for PKD in the future.

**Supplementary Information:**

The online version contains supplementary material available at 10.1186/s12864-022-08685-4.

## Background

Proliferative kidney disease (PKD) is an important disease of salmonids that has both economic and ecological significance. PKD is quite prevalent across Europe and North America that affects both wild salmonid population and domestic salmonid aquaculture [1]. PKD is caused by an evolutionarily degenerated cnidarian myxozoan endoparasite *Tetracapsuloides bryosalmonae* [2]. The two hosts parasitic life-cycle of *T. bryosalmonae* involve salmonid and bryozoans as intermediate and definitive hosts respectively [3, 4]. Climate change driven warmer water temperature favours the spread of *T. bryosalmonae* and increases the severity of PKD in the affected fish [5]. *T. bryosalmonae* infection along with warmer water temperature is responsible for the diminishing brown trout (*Salmo trutta*) population in the streams of the Alps [6]. A massive fish kill event, mainly mountain whitefish (*Prosopium williamsoni*) in the Yellowstone River, Montana, USA was caused due to *T. bryosalmonae* that led to the subsequent temporary lockdown of the river for public access followed by a huge loss in the associated recreational industry [7].

The portal entry of *T. bryosalmonae* into the salmonid host is through gills and eventually, the parasite reaches various internal organs like the kidney, spleen, and liver [8]. However, the kidney is the primary organ for infection where the parasite undergoes extrasporogonic development and is subsequently released via urine [8]. The released parasite can readily infect the bryozoan host. The development of *T. bryosalmonae* in the affected fish causes a chronic inflammatory reaction leading to the swelling of the kidney and spleen [1]. Interestingly, the European strain of *T. bryosalmonae* can infect and cause PKD in rainbow trout (*Oncorhynchus mykiss*), brown trout, and brook trout (*Salvelinus fontinalis*). However, the mature spores of the parasites can be released only by native brown trout and brook trout, whereas the exotic rainbow trout remains as a dead-end host [9]. Moreover, brown trout can serve as a reservoir host in which the parasite can persist and get released even after five years post-recovery from the clinical signs of PKD [10].

Due to the PKD associated huge economic losses in the rainbow trout aquaculture industry, most of the laboratory experiments to understand the host-pathogen interaction of this disease was done in the dead-end rainbow trout host [11]. In rainbow trout, PKD is manifested by dysregulated proliferation of B lymphocytes, hyper-secretion of immunoglobulins, imbalance in the cytokines of T-helper-like cells and up-regulation of SOCS genes [12–18]. However, few experiments were also done in brown trout to understand the host response during PKD at the molecular level suggests increased B cell response and higher Th1- like cytokine production [17, 19–22].

Alternative splicing is a post-transcriptional mechanism that gives rise to distinct multiple mRNAs from a single gene that may lead to proteins with a distinct function, stability, and sub-cellular localization [23]. Alternative splicing is observed in more than 90% of human genes [23]. Specific *cis*- and *trans*-regulatory elements control the inclusions of different exons spliced in the mature mRNA. Based on the splicing pattern alternative splicing can be classified into five basic types, (i) alternative 3′ splice site (A3SS), (ii) alternative 5′ splice site (A5SS), (iii) exon skipping (SE), (iv) intron retention (RI), and (v) mutually exclusive exons (MXE) [24]. Furthermore, abnormal mRNA transcripts having premature termination codons (PTCs) are eliminated by non-sense mediated decay [25]. Interestingly, about one-third of the spliced transcripts shall have such PTCs and are targeted for non-sense mediated decay [26].

RNA splicing increases the diversity of mRNA translated from the genome and thereby increases proteome diversity in eukaryotes. The protein isoforms formed due to alternative spliced gene variants may have different structural and functional properties [27]. Studies suggest that any alternation in the splicing pattern can affect the normal physiological state of the organism. In humans, chronic diseases like cancer and arthritis are associated with alternatively spliced genes. Furthermore, pathogens can alter the splicing pattern of the host during host-pathogen interaction [28]. The mechanism of alternative splicing is highly regulated and the dynamics of the splicing event changes under different physiological conditions [27]. Moreover, alternative splicing is known to regulate various molecular functions of an organism including immune response [29, 30] and disease resistance [31, 32]. Only a few studies are available in fish to understand the alternative splicing mechanism during host-pathogen interaction. Alternative splicing plays an important role in channel catfish (*Ictalurus punctatus*) during heat stress to increase the tolerance of the fish [33]. Furthermore, genes involved in the host defense mechanism were differentially expressed alternatively spliced (DEAS) in channel catfish during bacterial infections such as *Edwardsiella ictaluri* [34] and *Flavobacterium columnare* [33].

Recently, next-generation sequencing technology has made a substantial contribution in sequencing the chromosome level assembly of brown trout genome [35]. RNA-seq based transcriptomics has opened up avenues to explore differential gene expression, post-transcriptional modifications, and novel transcripts during host-pathogen interaction [36]. Furthermore, generating molecular information about the pathogen shall be valuable in developing efficient prophylactic measures [37]. RNA-seq has been employed to understand and generate the transcriptome resources of *T. bryosalmonae* [38, 39] and its hosts [21, 40–42]. However, there are no studies to explore the differentially expressed alternatively spliced transcripts in salmonids during proliferative kidney disease. In this study, we utilized the RNA-seq data generated in our previous experiment from the PKD-affected brown trout [21], to gain insights on the alternative splicing events during host-parasite interaction.

## Results

### Mapping of RNA-seq data

Relatively, a high number of interstitial pre-sporogenic stages of *T. bryosalmonae* in the posterior kidney of brown trout were observed using immunohistochemistry at 12 weeks post exposure (wpe) [21, 22]. Hence, the samples collected at 12 wpe were used for RNA-seq analysis. A total of 421.8 million raw 100 bp single-end RNA-seq reads were generated from the posterior kidney of brown trout (6 exposed and 6 controls) using Illumina platform. Subsequently, 420.6 million high quality reads were obtained after quality control and trimming. About 381.01 million reads (90.58%) mapped to the brown trout genome. A detailed summary of RNA-seq data and its mapping with the brown trout genome is provided in Supplementary File S1.

### Alternative splicing landscape in PKD affected brown trout

**Fig. 1:**
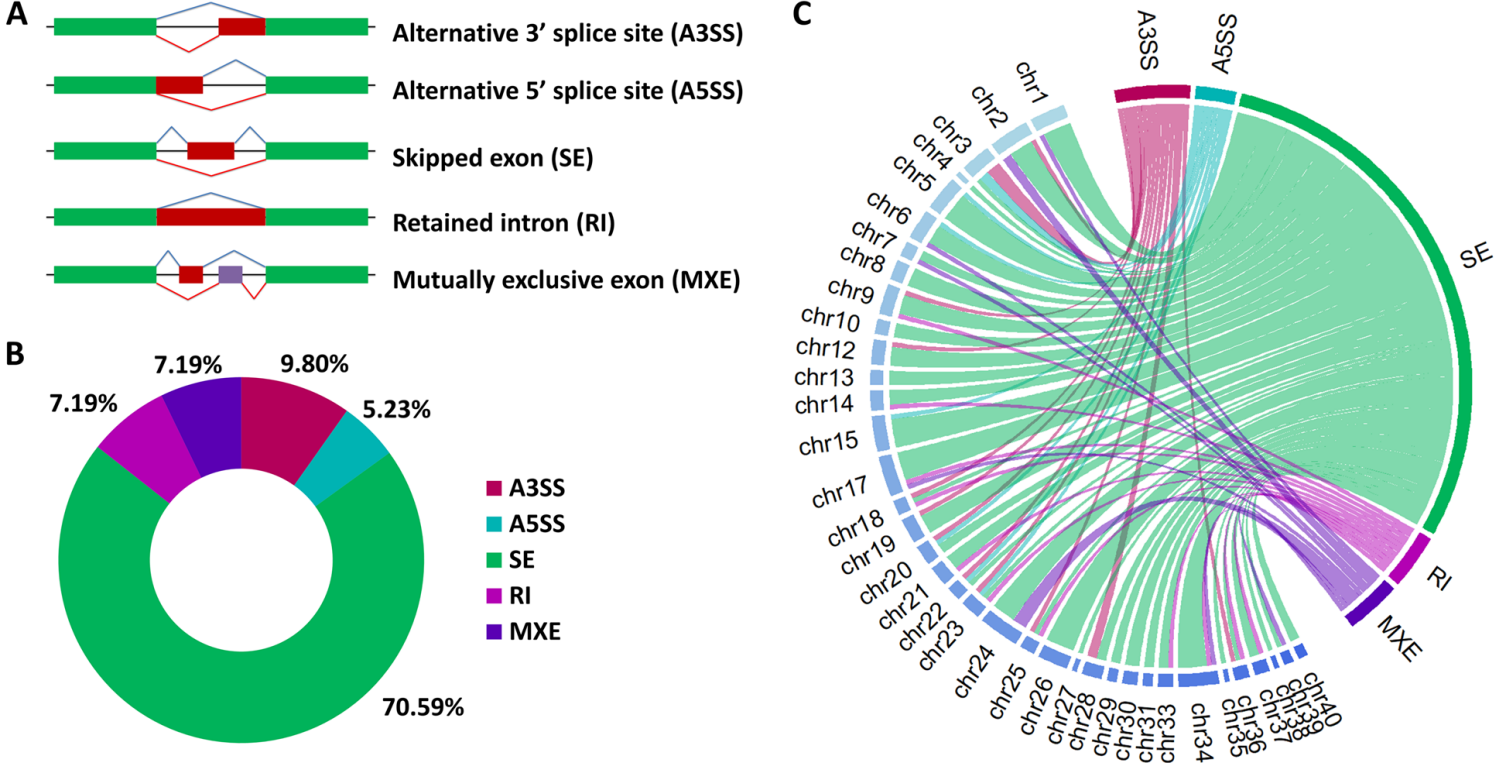
Alternative splicing landscape in the posterior kidney of PKD affected brown trout. **A** Illustration of five basic types of alternatively spliced events A3SS, A5SS, SE, RI, and MXE. **B** Schematic illustration of percentage abundance of significant alternative splicing events. **C** Chord diagram illustrating the chromosome-wise distribution of significant alternative splicing events in the brown trout genome

**Fig. 2:**
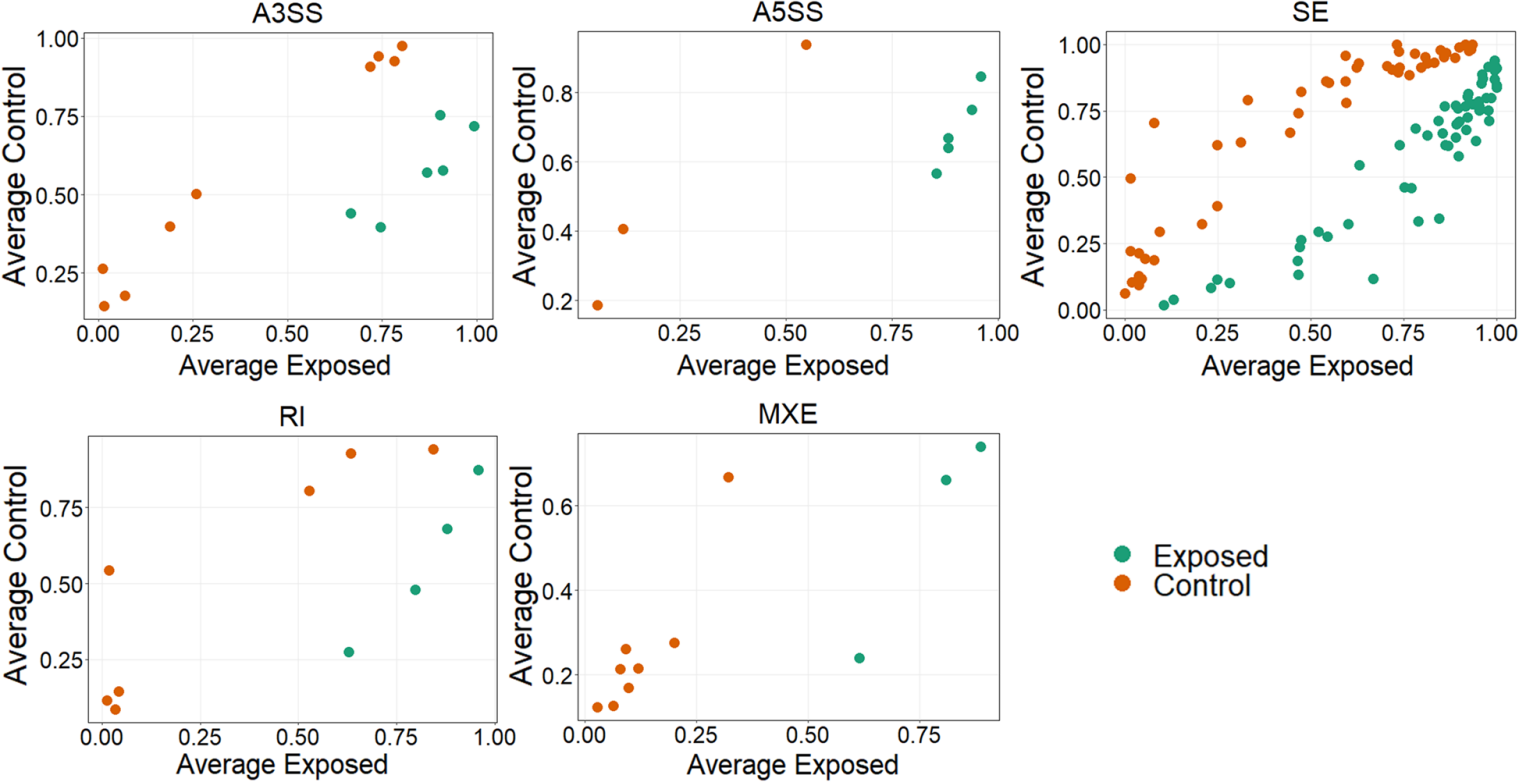
Scatter splicing plots of significant DEAS genes associated with proliferative kidney disease in brown trout. The plot displays mean PSI values with significant DEAS genes between exposed and control groups under five different splicing patterns A3SS (15), A5SS (8), SE (108), RI (11), and MXE (11)

**Fig. 3:**
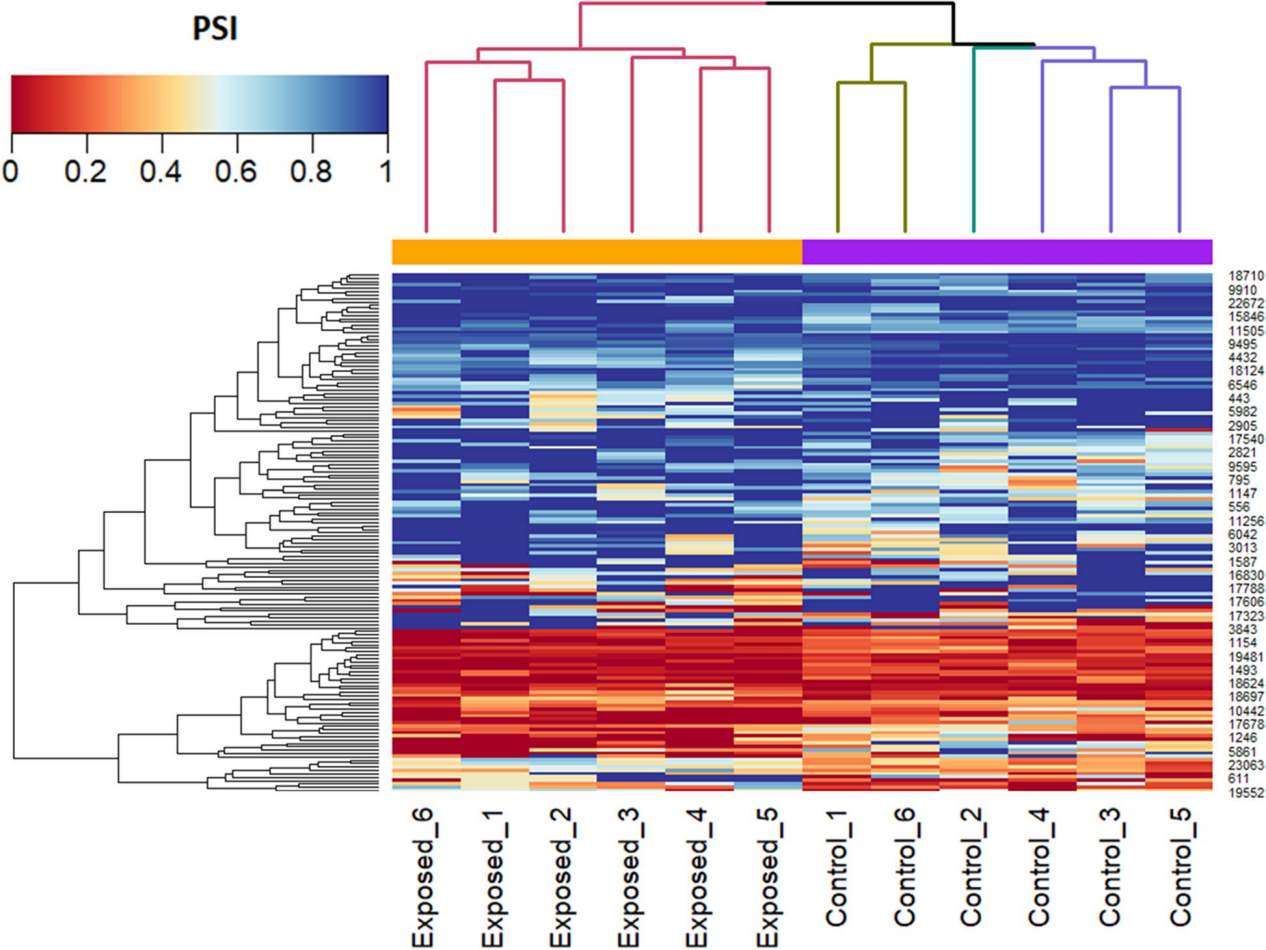
Heat map and hierarchical clustering of significant DEAS genes. The heatmap illustrates PSI values for the 153 significant DEAS genes were identified between exposed and control groups. Hierarchical clustering was constructed based on Euclidean distance and complete linkage. Each row corresponds to a single DEAS gene and each column corresponds to an individual posterior kidney sample

A total of 19,722 alternative spliced genes were differentially expressed in the posterior kidney of brown trout during PKD. Significant DEAS genes were filtered based on differential percent spliced-in (PSI) value and FDR *P*-value. About 153 alternative spliced genes were filtered and identified as significant DEAS genes (delta PSI = 5%, FDR *P-*value < 0.05) (Table 1). Moreover, out of these 153 DEAS genes, about 77 and 76 DEAS genes had increased and decreased inclusion levels, respectively (Table 1 and Supplementary File S2). All the five basic types of alternatively spliced events A3SS, A5SS, SE, RI, and MXE (Fig. 1A) were observed among the 153 DEAS genes. The most abundant DEAS event was SE (70.59%) followed by A3SS (9.8%), RI (7.19%), MXE (7.19%), and A5SS (5.23%) (Fig. 1B). The distribution of all the five basic types of alternatively spliced events in different chromosomes (chr) of brown trout during PKD is illustrated in Fig. 1C. The significant DEAS genes were distributed in all most every chromosome of brown trout except chr11, chr16, and chr32. Moreover, the highest DEAS gene distribution was observed in chr17, chr24, and chr34 with each chromosome having 8 events. The splicing plot of filtered 153 DEAS genes computed based on mean PSI values and categorised under all the five different splicing types is demonstrated in Fig. 2. Hierarchical clustering of individual PSI values of the 153 significant DEAS genes clustered the exposed and control groups separately (Fig. 3).

**Table 1:** Summary of alternative splicing events in the posterior kidney of PKD affected brown trout. The alternative splicing events were identified from the RNA-seq data generated at 12 wpe

Alternatively spliced Event	Over all events	Filtered events │Δ ψ│ > 5% FDR ***P-***value < 0.05	Increased inclusion alternatively spliced levels	Decreased inclusion alternatively spliced levels
A3SS	1528	15	6	9
A5SS	1394	8	5	3
SE	13,390	108	59	49
RI	1815	11	4	7
MXE	1595	11	3	8
Total	19,722	153	77	76

### Functional enrichment analysis of DEAS genes

**Fig. 4:**
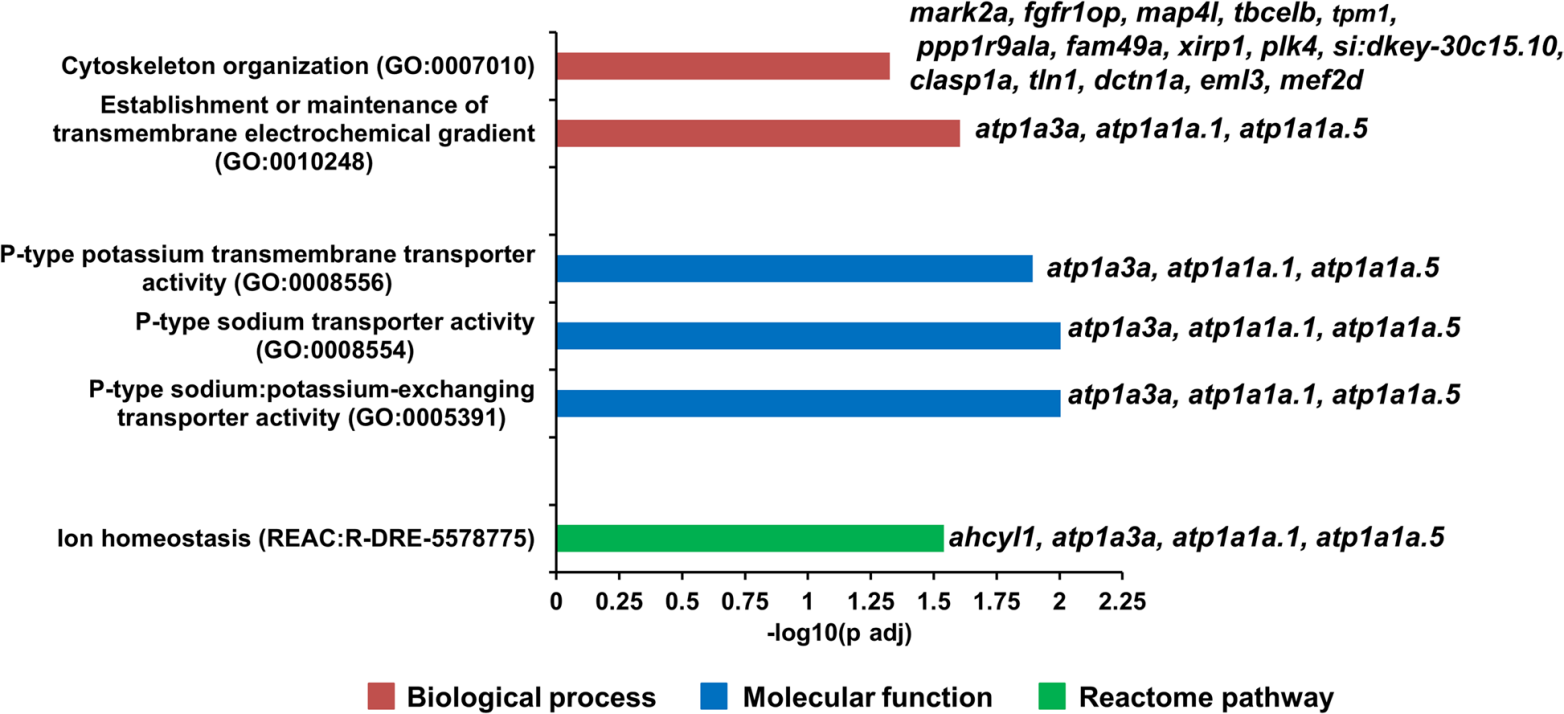
Functional enrichment of DEAS genes in the posterior kidney of *T. bryosalmonae* infected brown trout. The DEAS genes were statistically significantly enriched (adjusted *P-*value < 0.05) under the categories biological process, molecular function, and reactome pathway. The values of the bars under each category represent negative log_10_ of adjusted *P*-value

The orthologous Ensembl gene identifiers of zebrafish were identified for the significant DEAS genes of brown trout and used for functional enrichment analysis (Supplementary File S3). Overrepresentation or functional enrichment of DEAS genes in the PKD affected brown trout is illustrated in Fig. 4. The DEAS genes were significantly (adjusted *P-*value < 0.05) enriched for biological processes such as cytoskeleton organization (GO:0007010) and establishment or maintenance of transmembrane electrochemical gradient (GO:0010248). Furthermore, the DEAS genes were enriched for molecular function such as P-type sodium/potassium-exchanging transporter activity (GO:0005391), P-type sodium transporter activity (GO:0008554) and P-type potassium transmembrane transporter activity (GO:0008556). In the Reactome pathaway database the DEAS genes significantly enriched for Ion homeostasis (REAC:R-DRE-5578775) pathway. However, no statistically significant enrichment of DEAS genes was found under the categories of cellular components, and KEGG pathway database (www.kegg.jp/kegg/kegg1.html) [43].

### Protein-protein interaction

**Fig. 5:**
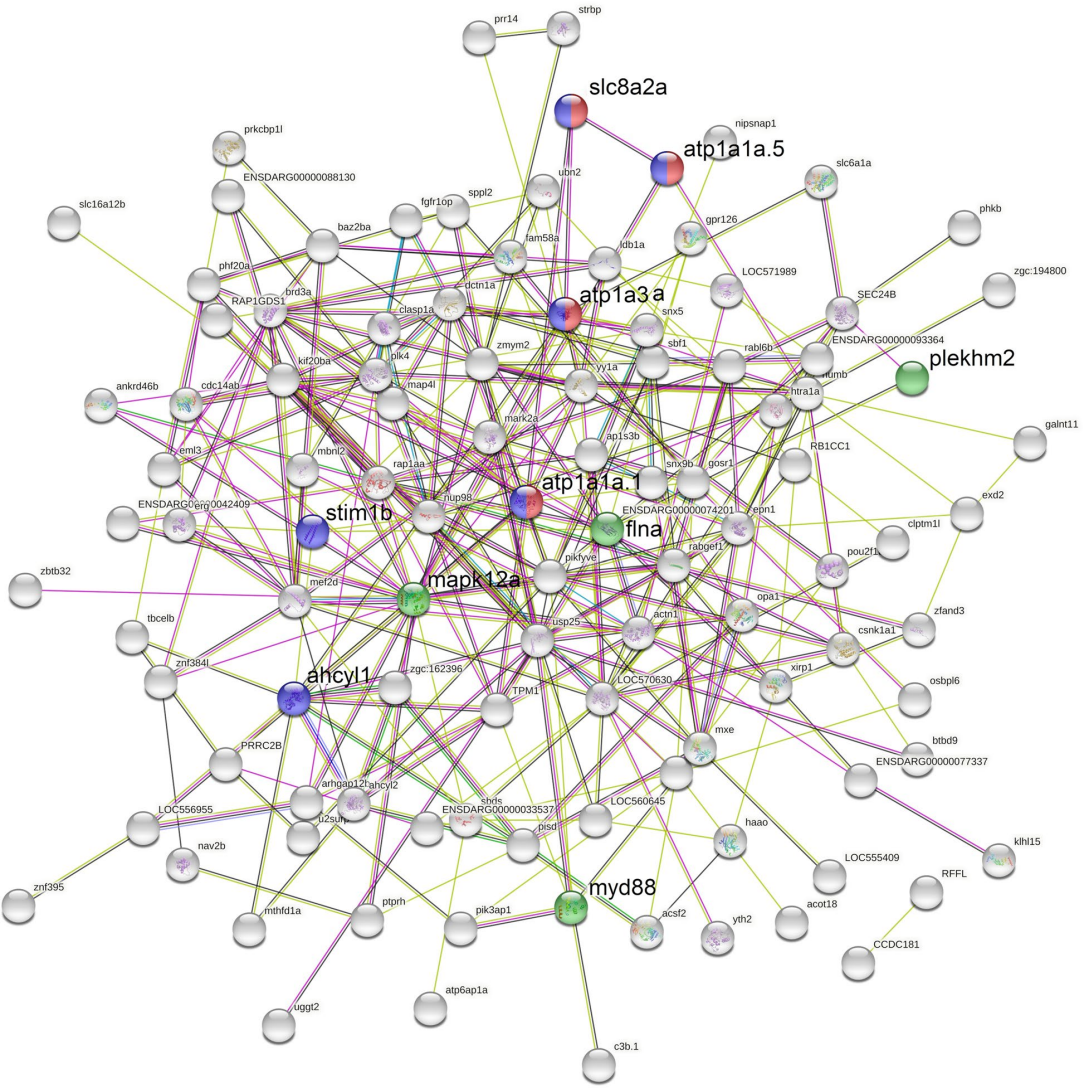
The protein-protein interaction network of DEAS genes in brown trout during PKD. Each node represents an individual protein and the interconnecting lines display the predicted functional interaction between the connected protein nodes. The numbers of lines are directly proportional to the strength of prediction for the functional association between proteins. Various sources of evidence were used to predict the association between proteins such as curated databases (light blue), experimentally determined (pink), gene neighbourhood (green), gene fusion (red), gene co-occurrence (dark blue), textmining (yellow) and co-expression (black). Proteins associated with local network clusters of Cation transporting ATPase, C-terminus, and Sodium / potassium ATPase beta chain (CL:20005), and mixed inclusion of Ion homeostasis, and EF-hand domain (CL:19893) are displayed in red and blue nodes respectively. Furthermore, proteins associated with Salmonella infection pathway (dre05132) is illustrated as a green node

**Fig. 6:**
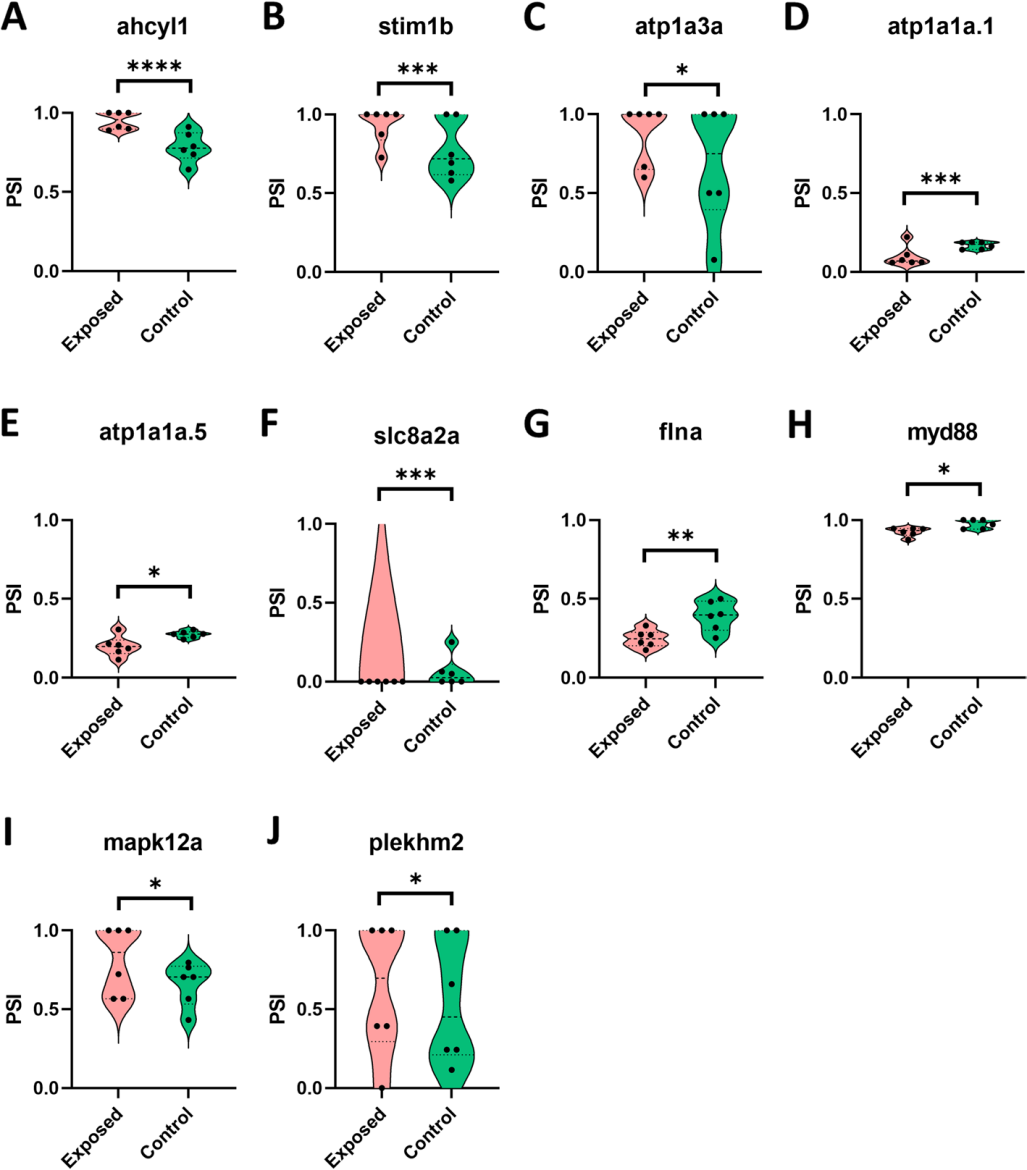
Violin plot illustrating PSI value of DEAS genes from brown trout exposed and control groups. The DEAS genes enriched in the protein-protein interaction network are displayed in the image, (**A)** ahcyl1, (**B)** stim1b, (**C)** atp1a3a, (**D)** atp1a1a.1, (**E)** atp1a1a.5, (**F)** slc8a2a, (**G)** Flna, (**H)** myd88, (**I)** mapk12a, and (**J)** plekhm2. Where, * - FDR *P* value < 0.05; ** - FDR P value < 0.01; *** - FDR P value < 0.001; and **** - FDR P value < 0.0001

String database assigned 116 (75.8%) out of 153 DEAS genes identified in the posterior kidney of brown trout during PKD for the construction of a protein-protein interaction network (Fig. 5). There were 116 nodes and 302 edges in the protein-protein interaction network. The network had an average node degree of 5.21 and an average clustering coefficient of 0.326 (enriched *P*-value = 0.000741). The protein-protein interaction network demonstrated Ubiquitin-specific peptidase 25 (USP25) as the central node with 21 distinct interactions. Two local network clusters were predicted to be significantly enriched, (i) Cation transporting ATPase, C-terminus, and Sodium / potassium ATPase beta chain (CL:20005, FDR *P*-value = 0.0089), and (ii) mixed inclusion of Ion homeostasis, and EF-hand domain (CL:19893, FDR *P*-value = 0.0047) (Table 2 and Fig. 6 A-F). In addition, the protein-protein interaction network was significantly enriched for Salmonella infection pathway (dre05132, FDR *P*-value = 0.0357) under KEGG pathways [43] (Table 2 and Fig. 6 G-J). Abbreviations of proteins used in the protein-protein interaction network are listed in Supplementary File S4.

**Table 2:** List of DEAS genes enriched in the protein-protein interaction network analysis. The inclusion level difference between exposed and control is computed in rMATS

Sl No	Gene code	Description	Splicing event	Inclusion level difference	Enrichment in protein-protein interaction network
1	ahcyl1	S-adenosylhomocysteine hydrolase-like protein 1	SE	0.165	Mixed inclusion of Ion homeostasis and EF-hand domain cluster (CL:19893)
2	stim1b	Stromal interaction molecule 1b	SE	0.159	Mixed inclusion of Ion homeostasis and EF-hand domain cluster (CL:19893)
3	atp1a3a	ATPase Na^+^/K^+^ transporting subunit alpha 3a	RI	0.198	Cation transporting ATPase C-terminus and Sodium/potassium ATPase beta chain cluster (CL:20005); Mixed inclusion of Ion homeostasis and EF-hand domain cluster (CL:19893)
4	atp1a1a.1	ATPase Na^+^/K^+^ transporting subunit alpha 1a tandem duplicate 1	MXE	−0.07	Cation transporting ATPase C-terminus and Sodium/potassium ATPase beta chain cluster (CL:20005); Mixed inclusion of Ion homeostasis and EF-hand domain cluster (CL:19893)
5	atp1a1a.5	ATPase Na^+^/K^+^ transporting subunit alpha 1a tandem duplicate 5	MXE	−0.075	Cation transporting ATPase C-terminus and Sodium/potassium ATPase beta chain cluster (CL:20005); Mixed inclusion of Ion homeostasis and EF-hand domain cluster (CL:19893)
6	slc8a2a	Solute carrier family 8 member 2a	SE	−0.061	Cation transporting ATPase C-terminus and Sodium/potassium ATPase beta chain cluster (CL:20005); Mixed inclusion of Ion homeostasis and EF-hand domain cluster (CL:19893)
7	flna	Filamin A alpha	SE	−0.143	Salmonella infection pathway (dre05132)
8	myd88	MYD88 innate immune signal transduction adaptor	SE	−0.058	Salmonella infection pathway (dre05132)
9	mapk12a	Mitogen-activated protein kinase 12a	MXE	0.148	Salmonella infection pathway (dre05132)
10	plekhm2	Pleckstrin homology domain containing family M	SE	0.087	Salmonella infection pathway (dre05132)

### Validation of DEAS genes

**Fig. 7:**
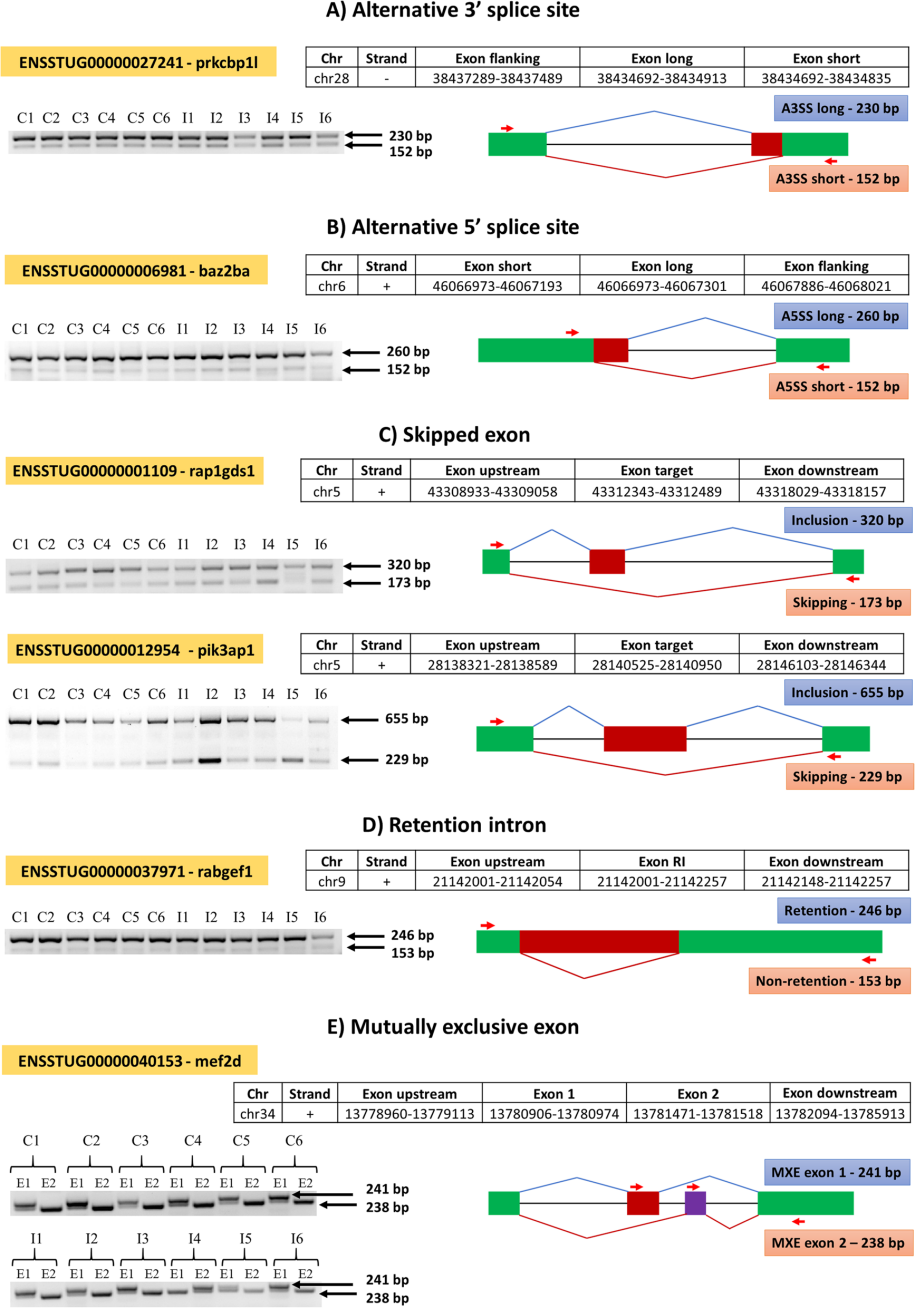
PCR Validation of selected DEAS gene. cDNA from control (C1 to C6) and infected (I1 to I6) samples were used for PCR and the amplified PCR product was visualized using agarose gel electrophoresis (The complete images of agarose gel electrophoresis are provided in the Supplementary File S6). In the transcript structure, the boxes (green, red, and purple) indicates exons, the straight black lines represent introns, the splice junctions are represented by flexed lines (blue and red), and the position of primers is illustrated by red arrows. The position of the transcripts and their splice sites in the brown trout genome is proved in the Table. **A** Alternative 3′ splice site is represented by ENSSTUG00000027241 - prkcbp1l, (**B)** Alternative 5′ splice site is represented by ENSSTUG00000006981 - baz2ba, (**C)** Skipped exon is represented by ENSSTUG00000001109 - rap1gds1 and ENSSTUG00000012954 - pik3ap1, (**D)** Retention intron is represented by ENSSTUG00000037971 - rabgef1, and (**E)** Mutually exclusive exon is represented by ENSSTUG00000040153 mef2d

The validation of a subset of the DEAS gene was done using reverse transcription PCR (RT-PCR). A3SS event was represented by protein kinase C binding protein 1 - like (prkcbp1l), and both A3SS long (230 bp) and A3SS short (152 bp) alternative spliced products were amplified (Fig. 7A). Bromodomain adjacent to zinc finger domain 2Ba (baz2ba) was validated as a representative of the A5SS event, and A5SS long (260 bp) and A5SS short (152 bp) alternative spliced products were amplified (Fig. 7B). SE event was represented by RAP1, GTP-GDP dissociation stimulator 1 (rap1gds1), and phosphoinositide-3-kinase adaptor protein 1 (pik3ap1) (Fig. 7C). The amplified PCR product size of the inclusion exon and skipped exon of rap1gds1 was 320 bp and 173 bp respectively. Similarly, pik3ap1 had a PCR product size of 655 bp (inclusion exon) and 229 bp (skipped exon). The alternative splicing event RI was represented by RAB guanine nucleotide exchange factor 1 (rabgef1) and both retention (246 bp) and non-retention (153 bp) isoforms were detected (Fig. 7D). The isoforms of myocyte enhancer factor 2d (mef2d) was validated as a representative of MXE alternative splice and both MXE exon 1 (241 bp) and MXE exon 2 (238 bp) were amplified (Fig. 7E). Furthermore, all the PCR products were sequenced, and the nucleotide sequences aligned exactly to their corresponding isoform sequences.

## Discussion

Fish are poikilothermic animals and their body temperature is constantly regulated by the temperature of the water. Furthermore, fish are constantly exposed to pathogens and other stressors in the aquatic environment. In order to cope with the unforeseeable environmental changes, evolutionarily fish might have developed a rapid regulatory molecular mechanism including post-transcriptional mechanism. Alternative splicing is a paramount post-transcriptional mechanism that regulates transcriptomics and proteomic diversity in all eukaryotic organisms [23]. The frequency of alternative splicing is inversely proportional to the genome size of the fish and hence the fishes having smaller genomes have to depend more on the alternative splicing mechanism for the synthesis of a diverse range of proteins [44]. The dynamics of the splicing pattern of an organism are highly influenced by environmental and physiological factors. Hence the splicing pattern and the functional role of spliced mRNA have always interested researchers. Moreover, RNA-seq based high throughput sequencing technologies have advanced the rapid identification and quantification of alternative splicing events [36]. Investigation of alternative splicing during host-pathogen interaction can provide biological insights about the host response during disease pathogenesis. However, only a few investigations are done in fish to understand the alternative splicing mechanism during infectious diseases, particularly during bacterial [34, 45] and viral [46] infections. To date, there are no studies in fish to understand the alternative splicing pattern during parasitic infection.

The kidney of teleost fish has both excretory and immune functions. In addition, the anterior kidney serves as a haematopoietic organ and its functions are synonymous with the functions of the bone marrow of higher vertebrates. Apart from these functions, the kidney of teleost fish has an important physiological role in ion homeostasis and osmoregulation [47]. Interestingly, *T. bryosalmonae* has been co-evolved with brown trout host and can undergo sporogenesis and persist for a long time in the kidney, and the parasitic spores are consistently shed via urine. In spite of the strong immune response, the *T. bryosalmonae* can proliferate in the kidney of the affected brown trout [20–22]. Hence kidney was chosen as the organ of interest for investigating the molecular response of brown trout during PKD.

The present study was aimed to investigate the alternative splicing mechanism in the posterior kidney of brown trout during PKD. The raw RNA-seq data generated from the PKD-affected brown trout from our previous experiment [21] and the recently published brown trout genomic sequence [35] were utilized in this study. In spite of the 19,722 DEAS genes identified during PKD pathogenesis in brown trout, only 153 were observed to be statistically significant. Moreover, we identified SE as the most common splicing pattern contributing 70.59% of the total DEAS event (Table 1 and Fig. 1). However, in the case of channel catfish, A3SS and A5SS were the most abundant DEAS event during *E. ictaluri* and *F. columnare* infections, respectively [34, 45].

Freshwater fish like brown trout dwells in a hyposmotic medium and the kidney of these animals have a major role in excreting osmotic water loads and re-absorption of filtered salts [48]. The development of *T. bryosalmonae* in the kidney of PKD affected brown trout may affect the ion homeostasis of the host. In the present investigation, DEAS genes involved in the ion homeostasis, particularly in the sodium/potassium transporter activity were significantly enriched (Fig. 4). They are S-adenosylhomocysteine hydrolase-like protein 1 (ahcyl1), ATPase Na^+^/K^+^ transporting subunit alpha 3a (atp1a3a), ATPase Na^+^/K^+^ transporting subunit alpha 1a tandem duplicate 1 (atp1a1a.1), and ATPase Na^+^/K^+^ transporting subunit alpha 1a tandem duplicate 5 (atp1a1a.5).

AHCYL1 is a novel enzyme that belongs to the AHCY family of proteins responsible for the metabolism of S-adenosyl-L-homocysteine [49]. Furthermore, AHCYL1 is involved in the inositol phospholipid signaling pathway and binds with inositol 1,4,5-trisphosphate receptor facilitating intracellular calcium (Ca^2+^) release leading to the activation of Ca^2+^ signalling [49]. Various biological processes such as organogenesis, fertilization, and apoptosis are regulated by AHCYL1 and inositol 1,4,5-trisphosphate receptor-mediated Ca^2+^ signaling cascade. Experiments in zebrafish suggest that AHCYL1 has a significant function in the embryogenesis of zebrafish [49]. Regulation of intracellular Ca^2+^ is important for the homeostasis of an organism and the differential regulation of AHCYL1 splice event in the present study may be a regulatory mechanism adapted by the brown host to attain homeostasis during PKD.

Sodium-Potassium ATPase is an enzyme protein present in the cell membrane of almost every cell of an animal. Moreover, this protein is highly conserved across fish and higher vertebrates [50]. Across the cell membrane, the sodium-potassium ATPase protein facilitates the transportation of three sodium (Na^+^) ions out and two potassium (K^+^) ions into the cell with a single molecule of ATP hydrolysis [51]. This Na^+^ / K^+^ transport has a crucial physiological function of ion homeostasis, transmembrane electrochemical Na^+^ gradient, nutrients transport across the cell, and cell signalling [52]. Moreover, sodium-potassium ATPase is important for regulating the resting potential and volume of the cell. Sodium-Potassium ATPase protein is a heterodimer having alpha and beta subunits [53]. In the present experiment, three alpha subunits (atp1a1a.1, atp1a1a.5, atp1a3a) were identified as DEAS genes during PKD pathogenesis in the kidney of brown trout. In zebrafish, a mutation in the atp1a1a.1 resulted in a slower functioning rate of the heart [54]. Moreover, knockdown of atp1a3a in zebrafish led to dilation in the ventricle of the brain and abnormal motility [55]. Hence sodium-Potassium ATPase is crucial for the normal physiology and biological functioning of all animals including fish.

In our previous study, transcriptome analysis in the posterior kidney of infected brown trout suggests that upregulated differentially expressed genes were enriched for the immune system process (58.54%) in the gene ontology analysis [21]. However, in the present study, immune-related DEAS genes were not statistically enriched in the gene ontology analysis. Alternatively, sodium-potassium transporter and ion homeostasis were enriched in the gene ontology analysis of DEAS genes in the posterior kidney of brown trout during PKD. It is known that the kidney is responsible for maintaining relatively constant levels of key ions including sodium, potassium, and calcium. It is possible that the molecular function of sodium-potassium transporter activity and ion homeostasis of the kidney might have been affected in the PKD affected brown trout. Moreover, alternative splicing of genes related to sodium-potassium transporter activity and ion homeostasis may be a regulatory mechanism to maintain homeostasis [56] in the PKD affected brown trout. However, further studies are needed to test this hypothesis.

In the protein-protein interaction network analysis two local network clusters, namely cation transporting ATPase C-terminus and Sodium/potassium ATPase beta chain cluster (CL:20005), and mixed inclusion of Ion homeostasis and EF-hand domain cluster (CL:19893) were significantly enriched (Table 2, Figs. 5 and 6 A-F). This is similar to the results of functional enrichment analysis of DEAS genes where genes related to ion homeostasis were significantly enriched in the PKD affected brown trout (Fig. 4). Furthermore, proteins involved in the salmonella infection pathway such as filamin A alpha (flna), MYD88 innate immune signal transduction adaptor (myd88), mitogen-activated protein kinase 12a (mapk12a), and pleckstrin homology domain containing family M (plekhm2) were significantly enriched in the network analysis (Table 2 and Fig. 6 G-J). Moreover, it is important to highlight that the protein-protein interaction network is predicted based on the functional interaction of zebrafish proteins. Hence, this network analysis warrants further detailed investigation.

## Conclusion

In conclusion, in this experiment, we determined the alternative splicing profile in the posterior kidney of brown trout during PKD. This is the first study in fish to explore the alternative splicing profile of the host during parasitic infection. Interestingly, genes involved in ion homeostasis were significantly enriched among the DEAS genes. Alternative splicing may have a vital role in maintaining the homeostasis of brown trout during the parasite development in the kidney. This study provides new biological insights into the alternative splicing pattern during PKD pathogenesis in brown trout. Of late, the importance and role of alternative splicing during host-pathogen interaction is being slowly recognized for developing therapeutic strategies. Hence, the insights gained from the present study can help to develop treatment measures for PKD.

## Methods

### Experiment and transcriptome data

This is a continuation of our previous work, where brown trout experimental infection and RNA-seq data generation are explained in detail [21]. Briefly, specific pathogen-free brown trout were exposed with *T. bryosalmonae* spores. The posterior kidneys from the exposed and non-exposed control groups were collected at 2, 4, 6, 8, 10, 12, and 17 wpe. The posterior kidney samples were fixed in RNAlater (Sigma, Steinheim, Germany) and 10% neutral-buffered formalin (NBF) for further studies. RNA extraction was done using RNeasy Mini Kit (Qiagen, Hilden, Germany) and 12 cDNA libraries were prepared from the posterior kidney of *T. bryosalmonae* exposed brown trout (exposed group, *n* = 6) and non-exposed control brown trout (control group, *n* = 6) at 12 wpe using TruSeq RNA Library Prep Kit v2 (Illumina, San Diego, CA, USA). Subsequently, the sequencing of cDNA libraries (100-bp single reads) was performed in Illumina Hi-seq 2500 platform. The raw reads obtained were deposited in the NCBI database under the bioproject ID PRJNA542491. The animal experiment was conducted in accordance with the regulations and guidelines of the §26 Austrian Law for Animal Experiments, Tierversuchsgesetz 2012 with permit number BMWFW-68.205/0181-WF/V/3b/2017. All methods of this study are reported in accordance to the ARRIVE guidelines for animal research [57].

### Mapping and alignment of RNA-seq reads

The NGS data was processed in the Galaxy server (https://usegalaxy.org/) [58]. The raw reads were assessed for quality using the tool FastQC (version 0.11.8). Adapter removal and quality trimming were done using Trimmomatic [59]. The trimmed reads were mapped to the brown trout genome (fSalTru1.1., ftp://ftp.ensembl.org/pub/release-100/fasta/salmo_trutta/dna/Salmo_trutta.fSalTru1.1.dna_sm.toplevel.fa.gz) obtained from Ensembl database using the splice aware mapping tool TopHat2 (Galaxy Version 2.1.1) [60]. Following this Cufflinks 2.2.1.3 tool was used to assemble the mapped reads to the transcripts [61].

### Alternative splicing analysis

The DEAS events between *T. bryosalmonae* exposed and control kidney were identified using rMATS turbo version 4.1.0 tool [62]. BAM files obtained from TopHat2 were provided as input with default settings in rMATS. Significant DEAS events (delta PSI = 5%, FDR *P-*value < 0.05) were filtered using Maser package version 1.10.0 in R program. Chromosome-wise distribution of significant DEAS events in the brown trout genome during PKD was visualized using circlize package version 0.4.12 in R program. R programming tool version 4.1.0 was used for the data analysis.

### Functional enrichment analysis

Functional enrichment analysis of DEAS events was done in g:Profiler web server [63]. Initially the Ensembl gene identifiers of significant DEAS events of brown trout were converted to the corresponding orthologous Ensembl gene identifiers of the model organism zebrafish (*Danio rerio*) using g:Convert option. Subsequently, the converted gene identifiers were provided as input in g:GOSt tool for functional enrichment analysis (adjusted *P-*value < 0.05) for biological process, molecular function, and pathways.

### Protein–protein interaction analysis of DEAS events

The protein–protein interaction network of DEAS events was constructed using STRING software version 11.0.b [64]. The converted gene identifiers of DEAS events were given as queries against zebrafish in active interactive sources of textmining, experiments, databases, co-expression, neighbourhood, gene fusion and co-occurrence with an interaction confidence score of 0.15.

### RNA extraction and reverse transcription PCR

RT-PCR was used to validate six representative DEAS genes (prkcbp1l, baz2ba, rap1gds1, pik3ap1, rabgef1, and mef2d). Briefly, total RNA was extracted from the posterior kidney of brown trout (exposed and control *n* = 6) with an on-column DNase I digestion step using RNeasy Mini Kit (Qiagen, Hilden, Germany). Quantification and quality check of the extracted RNA was done using NanoDrop 2000 spectrophotometer (Thermo Fisher Scientific, Vienna, Austria). Subsequently, one μg of total RNA per sample was used as a template for cDNA synthesis using the iScript cDNA synthesis kit (Bio-Rad, Hercules, USA). For designing primers initially, the alternative spliced transcripts and the spliced sites were visualized in the brown trout genome using integrated genome browser (IGB) version 9.1.8 [65]. Subsequently, the nucleic acid sequences were mined from IGB and used as input in the Primer3Plus tool to design specific primers [66]. The details of the primers and their binding position are provided in Supplementary File S5 and Fig. 7 respectively. The PCR reaction contained 5.0 μL of 2x DreamTaq Green PCR master mix (Thermo Scientific), 0.5 μL of each primer (10 pmol/μL), 1.0 μL DEPC-treated sterile distilled water (Carl Roth, Karlsruhe, Germany), and 3.0 μL of 1:20-fold diluted cDNA. The PCR reaction was performed in C1000 Touch Thermal Cycler (Bio-Rad, München, Germany) under the conditions of initial denaturation at 95 °C for 5 min, 35 cycles of denaturation (95 °C for 30 s), annealing (60 °C for 30 s) and extension (72 °C for 30 s), and the final extension was done for 10 min at 72 °C. The PCR products were subjected to electrophorese using 2% agarose gel containing Gel Red Nucleic Acid Gel Stain (Thermo Scientific) and visualized using a Bio Imaging System (Bio-Rad, München, Germany). The PCR products were excised from the gel, purified, and sequenced from both 5′ and 3′ directions. Subsequently, the sequences were aligned to the corresponding DEAS transcript and the structures of the splice variants were validated.

## Supplementary Information

**Additional file 1.**

**Additional file 2.**

**Additional file 3.**

**Additional file 4.**

**Additional file 5.**

**Additional file 6.**

## Data Availability

All raw RNA-seq reads are available in the NCBI Short Read Archive (SRA) database under the Bioproject ID PRJNA542491.

## Abbreviations

A3SS: Alternative 3′ splice site
A5SS: Alternative 5′ splice site
AHCYL1: S-adenosylhomocysteine hydrolase-like protein 1
atp1a1a.1: ATPase Na^+^/K^+^ transporting subunit alpha 1a tandem duplicate 1
atp1a1a.5: ATPase Na^+^/K^+^ transporting subunit alpha 1a tandem duplicate 5
atp1a3a: ATPase Na^+^/K^+^ transporting subunit alpha 3a
baz2ba: Bromodomain adjacent to zinc finger domain 2Ba
Ca^2+^: Calcium
Chr: Chromosomes
DEAS: Differently expressed alternatively spliced
flna: Filamin A alpha
K^+^: Potassium
IGB: Integrated genome browser
map k12a: Mitogen-activated protein kinase 12a
mef2d: Myocyte enhancer factor 2d
MXE: Mutually exclusive exons
myd88: MYD88 innate immune signal transduction adaptor
Na^+^: Sodium
pik3ap1: Phosphoinositide-3-kinase adaptor protein 1
PKD: Proliferative kidney disease
plekhm2: Pleckstrin homology domain containing family M
prkcbp1l: Protein kinase C binding protein 1 – like
PSI: Percent spliced-in
PTCs: Premature termination codons
rabgef1: RAB guanine nucleotide exchange factor 1
rap1gds1: RAP1 GTP-GDP dissociation stimulator 1
RI: Retention intron
RT-PCR: Reverse transcription PCR
SE: Skipped exon
USP25: Ubiquitin-specific peptidase 25
wpe: Weeks post exposure
