# Supplementary material for: Genome-wide alternative splicing profile in the posterior kidney of brown trout (Salmo trutta) during proliferative kidney disease
Source: BMC Genomics. 2022 Jun 16;23:446. doi: 10.1186/s12864-022-08685-4 (PMC9204890; doi:10.1186/s12864-022-08685-4)
Supplement: Supplementary file 5 — Additional file 5. [file 12864_2022_8685_MOESM5_ESM.docx]

| **Alternative 3’ splice site (A3SS)** | | | | |
| --- | --- | --- | --- | --- |
| **Ensembl ID & Gene** | **Primer ID** | **Sequence (5'->3')** | **A3SS short product length**  **(bp)** | **A3SS long product length**  **(bp)** |
| ENSSTUG00000027241  prkcbp1l | Prkc_F | AGGCACTTGGCATGGTACAC | **152** | **230** |
|  | Prkc_R | AGCCCCCTGAAGAAGAAGAA |  |  |
| **Alternative 5’ splice site (A5SS)** | | | | |
|  |  |  | **A5SS short product length**  **(bp)** | **A5SS long product length**  **(bp)** |
| ENSSTUG00000006981  baz2ba | Baz2_F | CCTCTGGACATAGCGGTGAC | **152** | **260** |
|  | Baz2_R | AGCTGCTGGAGACACATGG |  |  |
| **Skipped exon (SE)** | | | | |
|  |  |  | **Skipping Product (bp)** | **Inclusion product (bp)** |
| ENSSTUG00000001109  rap1gds1 | RAP1_F | GCTCCACTCCAAGGACCAC | **173** | **320** |
|  | RAP1_R | TGAGGCACATCTCTGTGAGG |  |  |
| ENSSTUG00000012954  pik3ap1 | Pik3_F | GCAGGGGAGTTATTTGGTGA | **229** | **655** |
|  | Pik3_R | TTCTGTAGCTTCCGCCTCAT |  |  |
| **Retention intron (RI)** | | | | |
|  |  |  | **Skipping Product length**  **(bp)** | **Intron**  **Inclusion product**  **(bp)** |
| ENSSTUG00000037971  rabgef1 | Rabg_F | TAATCGAGATGGATGCCAAG | **153** | **246** |
|  | Rabg_R | TGAAGTAGTAGCCACTCTCTCCTG |  |  |
| **Mutually exclusive exons (MXE)** | | | | |
|  |  |  | **MXE Exon1**  **(bp)** | **MXE Exon2**  **(bp)** |
| ENSSTUG00000040153 mef2d | Mef2_E1_F | ACAGAGCGGGGATTTGTCTA | **241** | |
|  | Mef2_R | CTCCAGGCACAGTAGGGAAC |  |  |
|  |  |  |  |  |
|  | Mef2_E2_F | CCAACCTCCTCTTGGGTAGA | **238** | |
|  | Mef2_R | CTCCAGGCACAGTAGGGAAC |  |  |

**Table S5.** List of primers used in this study to validate alternative splice events
