# Supplementary material for: Genome-wide alternative splicing profile in the posterior kidney of brown trout (Salmo trutta) during proliferative kidney disease
Source: BMC Genomics. 2022 Jun 16;23:446. doi: 10.1186/s12864-022-08685-4 (PMC9204890; doi:10.1186/s12864-022-08685-4)
Supplement: Supplementary file 6 — Additional file 6. [file 12864_2022_8685_MOESM6_ESM.docx]

**Table S6.** Complete images of the agarose gel electrophoresis of PCR amplicons illustrated in the figure 7 of the manuscript.

**A. Alternative 3’ splice site**

**prkcbp1l** (ENSSTUG00000027241): 100 bp – Marker; Prkc_C1 to C6 – Control samples; Prkc_I1 to I6 – Infected samples; Prkc_NC – Negative control (without DNA template).


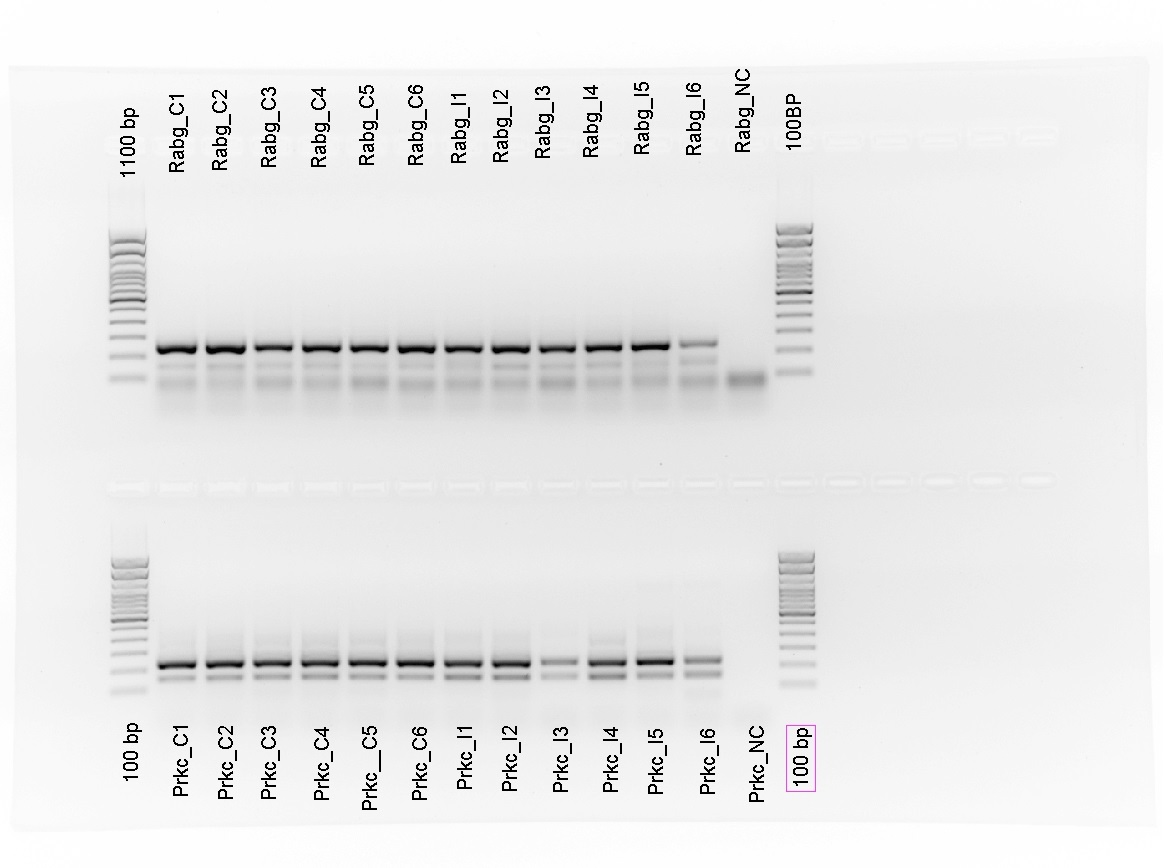


152 bp

230 bp

**B. Alternative 5’ splice site**

**baz2ba** (ENSSTUG00000006981): 100 bp – Marker; BAZ2_C1 to C6 – Control samples; BAZ2_I1 to I6 – Infected samples; BAZ2_NC – Negative control (without DNA template).


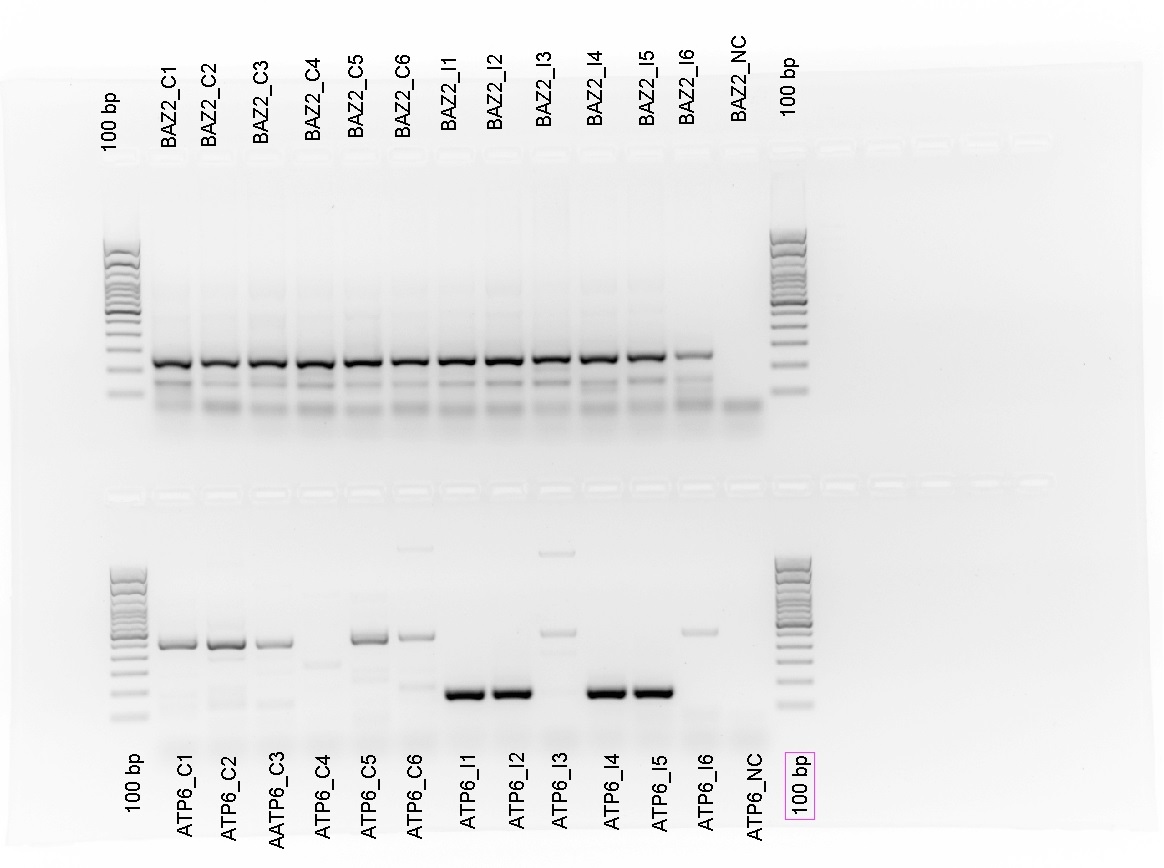


260bp

152 bp

**C. Skipped exon**

**Rap1gds1** (ENSSTUG00000001109): 100 bp – Marker; RAP1_C1 to C6 – Control samples; RAP1_I1 to I6 – Infected samples; RAP1_NC – Negative control (without DNA template).


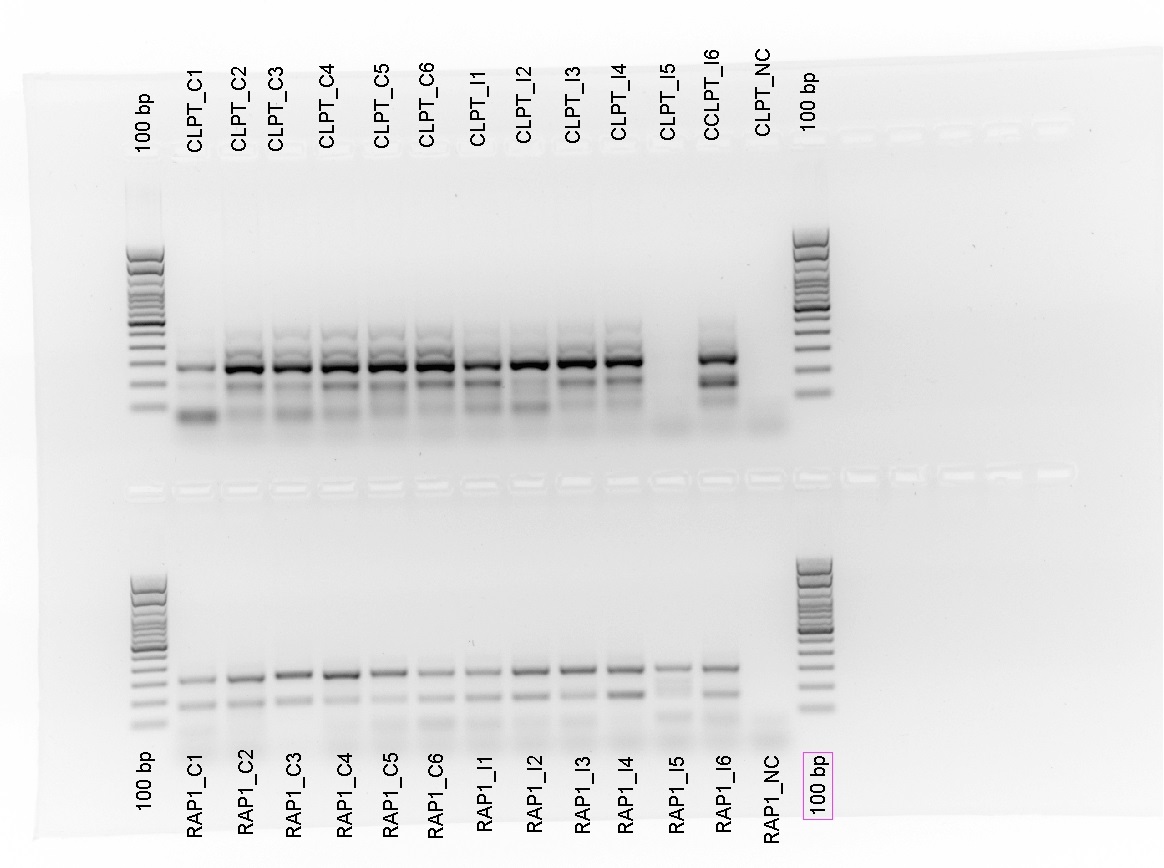


320bp

173 bp

**pik3ap1** (ENSSTUG00000012954): 100 bp – Marker; PIK3_C1 to C6 – Control samples; PIK3_I1 to I6 – Infected samples; PIK3_NC – Negative control (without DNA template).


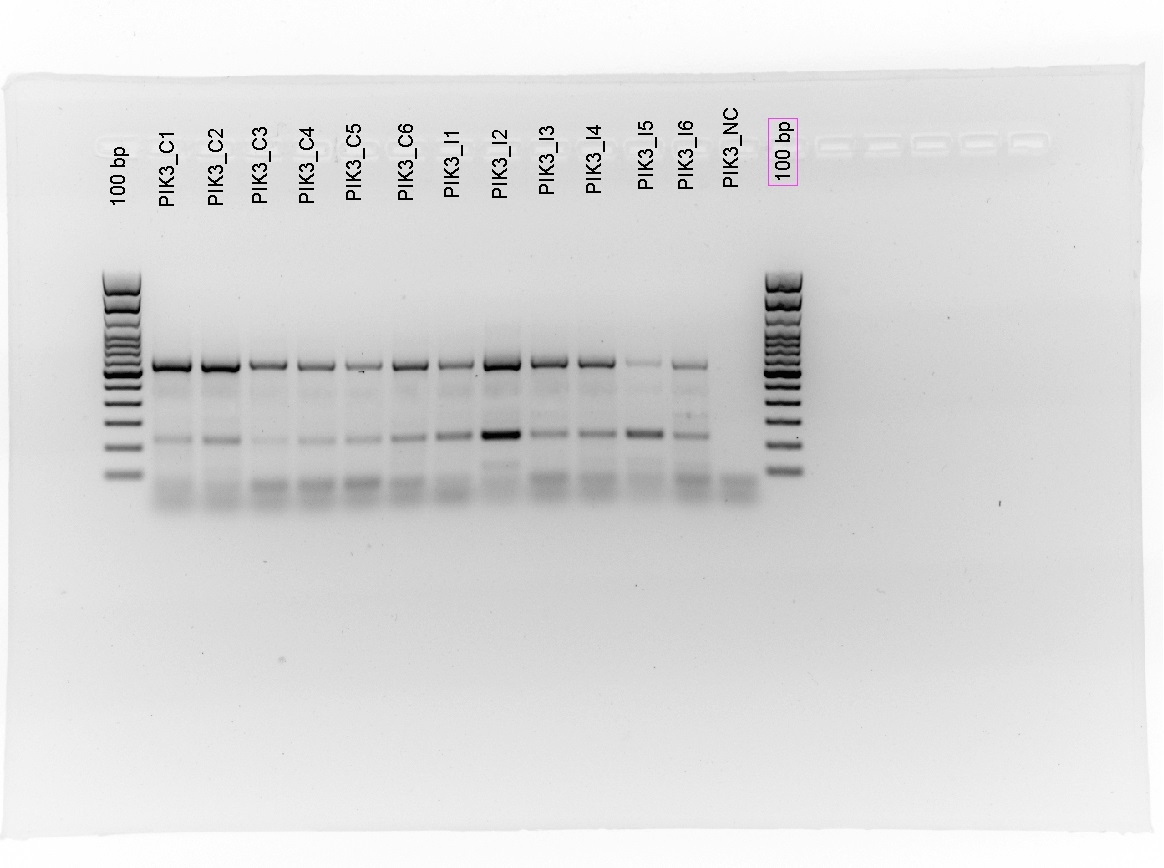


229 bp

655 bp

**D. Retention intron**

**rabgef1** (ENSSTUG00000037971): 100 bp – Marker; Rabg_C1 to C6 – Control samples; Rabg_I1 to I6 – Infected samples; Rabg_NC – Negative control (without DNA template).


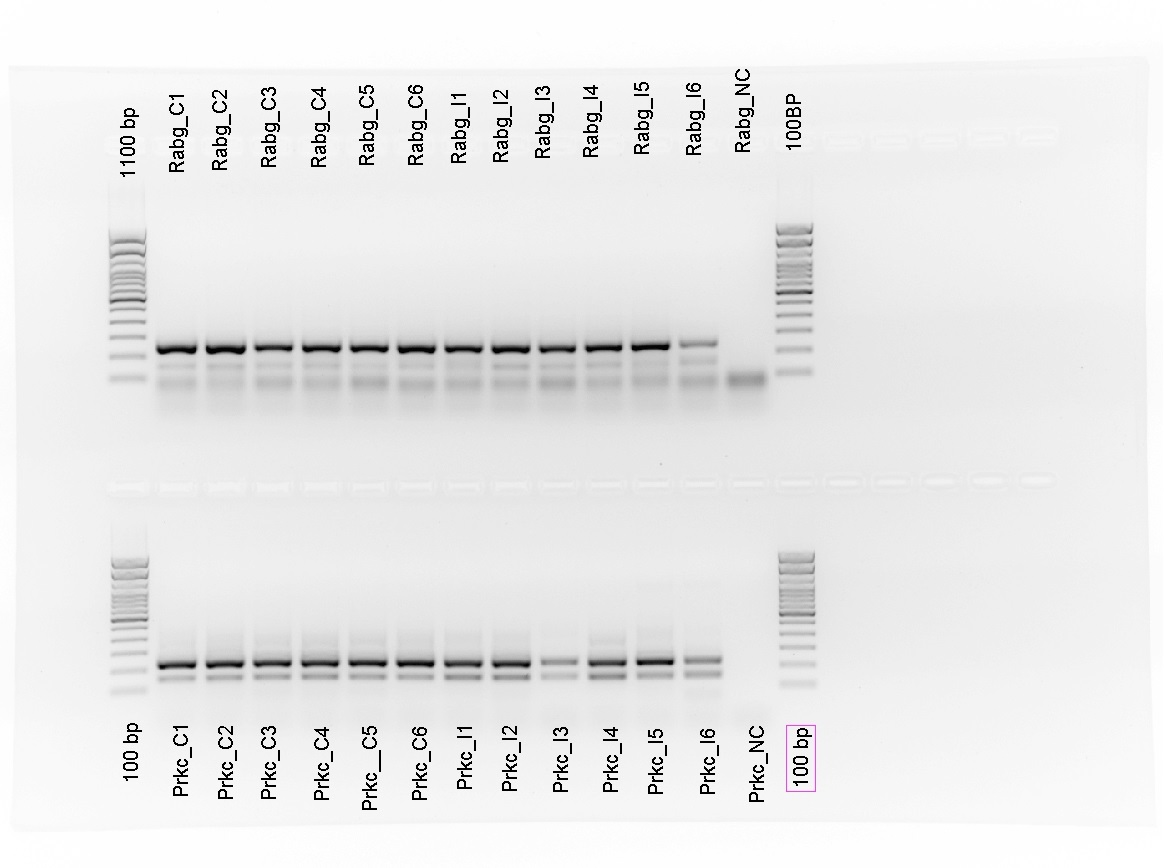


246 bp

153 bp

**E. Mutually exclusive exon**

**mef2d** (ENSSTUG00000040153): 100 bp – Marker; MEF-E1_C1 to C6 – Control samples targeting MXE exon 1; MEF-E2_C1 to C6 – Control samples targeting MXE exon 2; MEF-E1_I1 to I6 – Infected samples targeting MXE exon 1; MEF-E2_I1 to I6 – Infected samples targeting MXE exon 2; MEF-E1_NC – Negative control (without DNA template) for MXE exon 1; MEF-E2_NC – Negative control (without DNA template) for MXE exon 2.


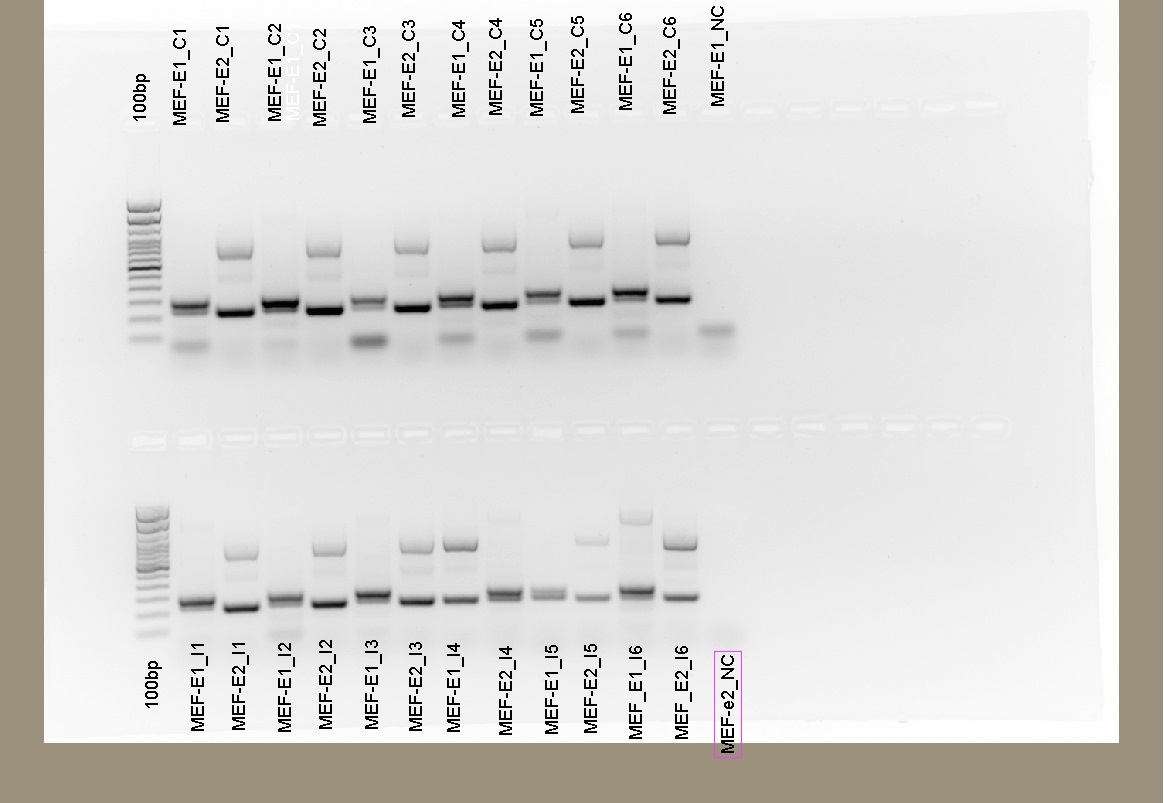


241 bp

238 bp

238 bp

241 bp
